# A designed fusion tag for soluble expression and selective separation of extracellular domains of fibroblast growth factor receptors

**DOI:** 10.1038/s41598-021-01029-4

**Published:** 2021-11-02

**Authors:** Dae-Eun Cheong, Hye-Ji Choi, Su-Kyoung Yoo, Hun-Dong Lee, Geun-Joong Kim

**Affiliations:** Department of Biological Sciences and Research Center of Ecomimetics, College of Natural Sciences, Chonnam National University, Yongbong-ro, Buk-gu, Gwangju, 61186 Korea

**Keywords:** Biotechnology, Microbiology

## Abstract

Fibroblast growth factor receptors (FGFRs) generate various transduction signals by interaction with fibroblast growth factors (FGFs) and are involved in various biological functions such as cell proliferation, migration, and differentiation. Malfunction of these proteins may lead to the development of various diseases, including cancer. Accordingly, FGFRs are considered an alternative therapeutic target for protein and/or gene therapy. However, the screening of antagonists or agonists of FGFRs is challenging due to their complex structural features associated with protein expression. Herein, we conducted the development of a protease-free cleavable tag (PFCT) for enhancing the solubility of difficult-to express protein by combining maltose-binding protein (MBP) and the C-terminal region of Npu intein. To validate the availability of the resulting tag for the functional production of extracellular domains of FGFRs (Ec_FGFRs), we performed fusion of PFCT with the N-terminus of Ec_FGFRs and analyzed the expression patterns. Almost all PFCT-Ec_FGFR fusion proteins were mainly detected in the soluble fraction except for Ec_FGFR4. Upon addition of the N-terminal region of Npu intein, approximately 85% of the PFCT-Ec_FGFRs was separated into PFCT and Ec_FGFR via intein-mediated cleavage. Additionally, the structural integrity of Ec_FGFR was confirmed by affinity purification using heparin column. Taken together, our study demonstrated that the PFCT could be used for soluble expression and selective separation of Ec_FGFRs.

## Introduction

The human fibroblast growth factor (hFGF) family consists of 22 members^1^. Except for intracrine FGFs (FGF11-14), which are not considered secreted proteins, most FGFs function as ligands that trigger various biological processes by establishing interaction with their cognate receptors, fibroblast growth factor receptors (FGFRs)^2^. FGFRs belong to the receptor tyrosine kinase family and consist of an extracellular domain containing three immunoglobulin-like domains (D1, D2, and D3), a single-pass transmembrane domain, and an intracellular protein-tyrosine kinase domain. FGFR1, 2 and 3 have isoforms generated via alternative splicing of the genes encoding these receptors^3^. FGFRs are expressed on the surface of different cell types. The specific interaction between FGFs and FGFRs is modulated by the extracellular domain, heparin sulfate (HS), and α/β-klotho. These interactions stimulate various signal transduction cascades that are implicated in embryonic development, homeostasis maintenance, cellular proliferation, migration, differentiation, and angiogenesis^4,5^.

Aberrant activation of FGFRs is associated with pathological conditions and the development of various cancers, including breast cancer, bladder cancer, liver cancer, and renal cell carcinoma^6–8^. Accordingly, FGFRs have gained considerable attention as promising therapeutic targets for several diseases. Recently, FGFR antagonists, including neutralizing antibodies^9–11^, have been developed and screened. In certain cases, the production of FGFRs with native folds, especially extracellular domains of FGFRs, is a critical step. However, thus far, extracellular domains of FGFRs have been obtained through refolding of inclusion bodies expressed from *Escherichia coli*^12^. Although the protein refolding technique can be applied to obtain many proteins, it lacks a universal refolding protocol, involves a labor-intensive process, and shows low reproducibility^13,14^.

**Figure 1:**
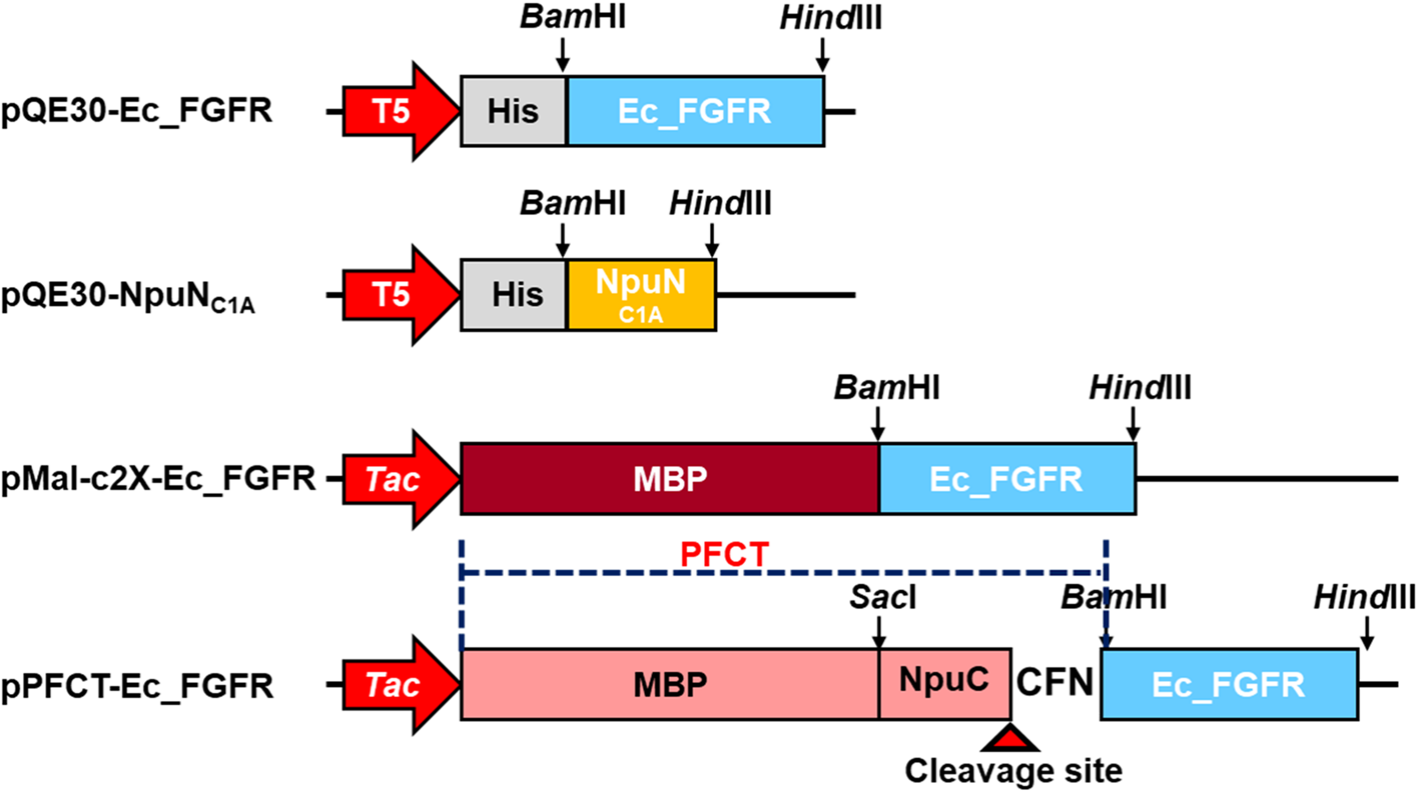
Recombinant DNA constructs used for the expression of protein of interest (POI) and protease-free cleavable tag (PFCT). In PFCT, NpuC consisted of 38 amino acids**,** including three additional residues CFN (+ 1 to + 3) of natural extein sequence. Therefore, three amino acids were additionally fused to the N-terminus of Ec_FGFRs after intein-mediated cleavage reaction.

We preliminarily observed that the expressed extracellular D2 and D3 domains of FGFRs (Ec_FGFRs) in insoluble form could be majorly converted into soluble form through maltose-binding protein (MBP) fusion at the N-terminus of Ec_FGFRs in *E. coli*, except for Ec_FGFR4. MBP was then removed from purified MBP-Ec_FGFRs via treatment with factor Xa protease; however, this step further resulted in the degradation of the cleaved Ec_FGFRs, and therefore, the separated protein Ec_FGFRs could not be obtained. To solve this problem, we conducted the development of the protease-free cleavable tag (PFCT) for enhancing the solubility of a protein of interest (POI) by combining MBP and the C-terminal region of DnaE split intein (Npu intein) derived from *Nostoc punctiforme* (Fig. 1). Subsequently, we performed fusion of PFCT with the N-terminus of Ec_FGFRs. Although the total expression level of PFCT-Ec_FGFR fusion proteins was slightly lower than that of only MBP fusion, the soluble expression ratio of fusion proteins was maintained. After separating from PFCT-Ec_FGFR by treating with the N-terminal region of Npu intein in vitro, the structural integrity of the resulting Ec_FGFRs was confirmed by affinity binding using a heparin column. To the best of our knowledge, this is the first report to show the soluble expression and selective separation of Ec_FGFRs without the necessity of refolding processes in *E. coli*. The finding of this study may facilitate the research and development of drugs for targeting FGFs and for treating FGFR-related diseases.

## Results and discussion

### Expression patterns and functional analysis of MBP-fused FGFRs

**Figure 2:**
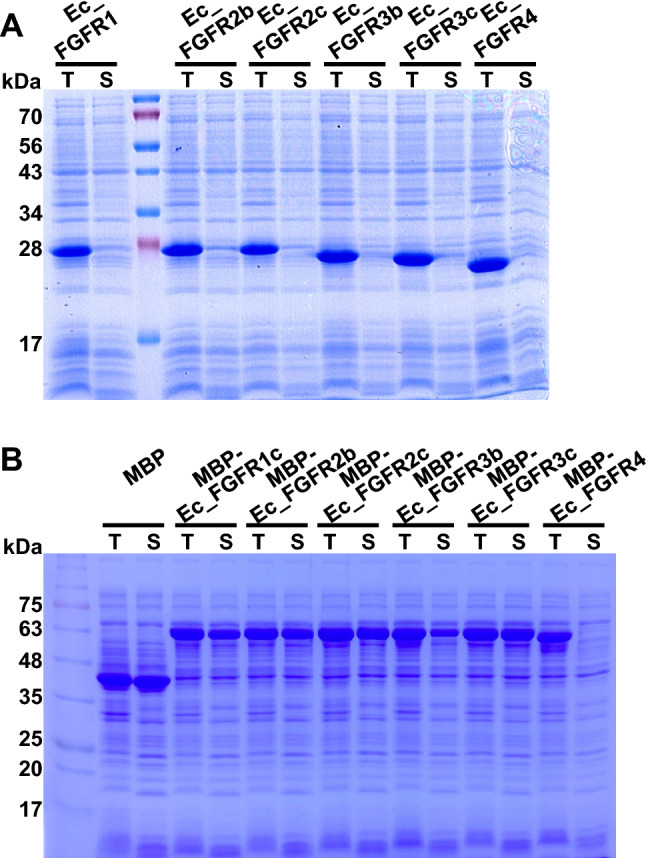
Analysis of 6× His- (**A**) and maltose-binding protein (MBP)-fused fibroblast growth factor receptor (Ec_FGFR) (**B**) proteins in *Escherichia coli* XL1-Blue via SDS-PAGE. All FGFR proteins (1c, 2b, 2c, 3b, 3c, and 4), consisting of minimal ligand-binding domains (D2 and D3) without D1 and D1–D2 linker, were expressed in *E. coli* XL1-Blue under the specified conditions described in the experimental section. T and S denote total and soluble fractions, respectively.

The extracellular domain of FGFRs typically consists of three immunoglobulin-like domains (D1, D2, and D3) and an acid box^15^. The D1 domain and acid box function as an autoinhibitory domain that regulates the overall conformation of FGFRs to avoid their autoactivation in the absence of their cognate growth factors^3,16^. The D2 and D3 domains are involved in the binding of FGFs to heparin sulfate and known as the minimal ligand-binding domains of FGFRs^17,18^. Based on these reports, we reasoned that the specific ligand-binding D2 and D3 domains could use for the screening and development of antagonists and/or drugs. However, these domains of FGFRs has a long disordered region (D142- K164 in FGFR1c) at the N-terminal region^19^. This structural feature may occasionally increase the susceptibility to proteolytic attack, a phenomenon which is closely associated with the high instability of these proteins. To counter the problematic properties of these minimal domains, the N-terminal region of FGFRs was further trimmed based on the predicted secondary structure (http://bioinf.cs.ucl.ac.uk/introduction/) and the deposited 3D structure in Protein Data Bank (https://www.rcsb.org/). Finally, FGFR1c (E162–E365), FGFR2b/c (E163–E369), FGFR3b/c (D160–E365), and FGFR4 (E166–D355) were selected (Ec_FGFRs) and cloned into pMAL-c2X to construct fusion proteins with MBP (Table S1). MBP is a well-known fusion partner of various proteins which exhibits function as an intrinsic molecular chaperone in the context of a MBP fusion protein, and results in the improvement of the solubility of its fusion partner^20^. Therefore, we preferentially expected that the solubility of Ec_FGFR could be enhanced by performing its fusion with MBP. As shown in Fig. 2, Ec_FGFRs were mainly expressed as soluble proteins through MBP fusion, except for Ec_FGFR4.

**Figure 3:**
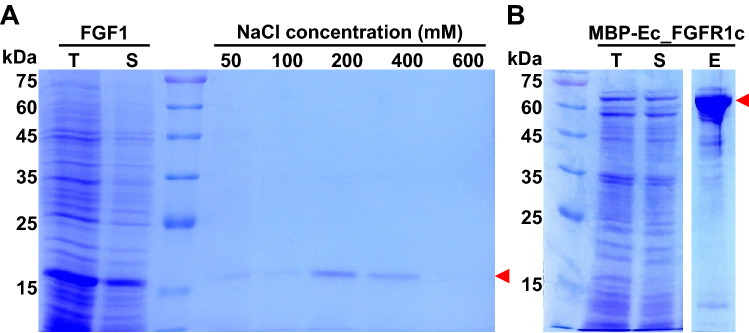
Purification of fibroblast growth factor 1 (FGF1) by receptor affinity using the amylose resin-bound maltose-binding protein (MBP)-fibroblast growth factor receptor 1c isoform (Ec_FGFR1c) protein. The soluble fraction of the recombinant *E. coli* BL21(D3) harboring pET24a-hFGF1 was loaded onto the column packed with amylose resin-bound MBP-Ec_FGFR1c. FGF1 was eluted from the resin using a stepwise gradient of NaCl (**A**) in repeated-batch mode. After all elution steps for FGF1 were completed, MBP-Ec_FGFR1 was finally stripped from amylose resin using 10 mM maltose in phosphate-buffered saline (PBS) (**B**). Red arrows indicate the target protein obtained. T, S and E denote total, soluble and elution fraction, respectively. The full length of gel image of Fig. 3B before image editing is shown in supplementary Fig. S1.

In the twofold symmetric dimer of the FGF1 and FGFR1c complex, each FGF1 interacted with a cognate receptor FGFR1c^21^ to enable the formation of a dimer for signal transduction. To preliminary test the ligand-binding activity and structural integrity of MBP-fused Ec_FGFR1c proteins, we aimed to recover FGF1 from the recombinant *E. coli* BL21(DE3) lysates by affinity chromatography using amylose resin that primarily bound to MBP-fused Ec_FGFR1c protein. As shown in Fig. 3 (see also the original Fig. S1), although weakly bound FGF1s were primarily eluted at concentrations of 50 and 100 mM NaCl, the main fraction of FGF1s was eluted from amylose resin-bound MBP-Ec_FGFR1c with NaCl at concentrations ranging between 200 and 400 mM. Despite the innate high affinity of Ec_FGFR1c for FGF1, FGF1 was eluted from the amylose resin-bound MBP-Ec_FGFR1c under conditions of relatively low salt concentrations. These results might be attributable to the steric hindrance occurring between Ec_FGFR1c and FGF1 induced by fused MBP and the absence of heparin sulfate. Heparin sulfate is known to enhance the binding of FGFs to FGFRs. Nevertheless, the resulting data showed that FGF1 could be purified from *E. coli* BL21(DE3) lysates by the specific binding to MBP-Ec_FGFR1c. This result also strongly indicated that the MBP-fused Ec_FGFR1c possessed suitable structural features for ligand binding.

### Expression patterns of PFCT-fused FGFR proteins

To separate Ec_FGFR1c from its fusion protein, the purified MBP-Ec_FGFR1c was treated with factor Xa protease according to the manufacturer’s instructions. However, enzymatic cleavages performed using this protease led to the degradation of almost all Ec_FGFR1c proteins (Fig. S2). As alternatives to this protease, other proteases such as thrombin and enterokinase are expensive and exhibit nonspecific cleavage patterns depending on the structure and topology around the cleavage site. To circumvent such hurdles, we performed the development of PFCT for enhancing the solubility of a POI by using fusion of MBP and the C-terminal region of DnaE split intein (Npu intein) originated from *N. punctiforme* (Table S2). Although protein purification and cleavage systems based on intein-mediated protein splicing have been previously reported and are commercially available^22–25^, most reported systems mainly use only a split intein as a fusion or tagging partner.

**Figure 4:**
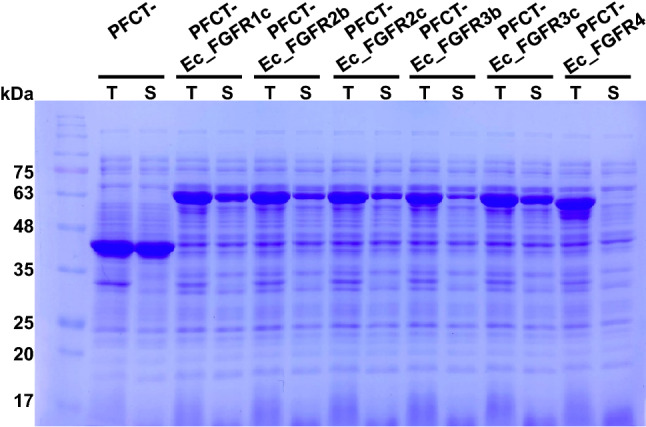
Expression patterns of protease-free cleavable tag (PFCT)-fibroblast growth factor receptor (Ec_FGFRs) fusion proteins in *E. coli* XL1-Blue. All constructed PFCT-Ec_FGFRs fusion proteins were expressed in *E. coli* XL1-Blue under the specified condition described in the experimental section. All clones showed appreciable growth under specified conditions and showed no distinct bands corresponding to the stress-related proteins in SDS-PAGE. T and S denote total and soluble fractions, respectively.

To combine the functions of enhanced solubility and protease-free cleavage, the C-terminal intein fragment consisting of 38 amino acids, including three additional residues CFN (+ 1 to + 3) of natural extein sequence^26,27^, was subjected to fusion at the C-terminus of MBP to serve as the recognition sequence for site-specific protein cleavage, leading to the generation of PFCT (Table S2). The solubility-enhancing function of PFCT was validated through the fusion of each Ec_FGFR to PFCT. When the expression patterns were compared between MBP- and PFCT-fused Ec_FGFRs, the expression levels of PFCT fusion proteins were observed to be slightly reduced; however, the ratio of total and soluble proteins did not change (Fig. 4). Exceptionally, Ec_FGFR4 was mainly expressed as inclusion bodies in the insoluble fraction. Based on the results obtained, we further confirmed whether functionally soluble Ec_FGFRs could be obtained through intein-mediated cleavage via addition of the N-terminal intein fragment.

### Removal kinetics of PFCT and verification of Ec_FGFRs

To validate the cleavage efficiency of PFCT, PFCT-fused Ec_FGFR1c was arbitrarily selected as a candidate and purified to apparent homogeneity for further analyses. The resulting purification yield of the fusion protein PFCT-Ec_FGFR1c was 23.8 mg/L. To trigger intein-mediated cleavage, the N-terminal fragment of *Npu* intein (His-NpuN_C1A_) was cloned into pQE30 where it was fused with a 6× His tag for purification at its N-terminus. During cloning, the first residue of the N-terminal fragment of NpuN was site-specifically mutated (C1A) to abrogate the N-terminal cleavage activity^28^ by PCR with a specific primer. The resulting construct was subcloned and analyzed using SDS-PAGE. The major portion of the expressed His-NpuN_CIA_ from pQE30 was monitored as insoluble aggregates; however, the yield (13 mg/L) and the purity achieved after purification were sufficient to cleave PFCT from PFCT-fused Ec_FGFR (Fig. S3).

**Figure 5:**
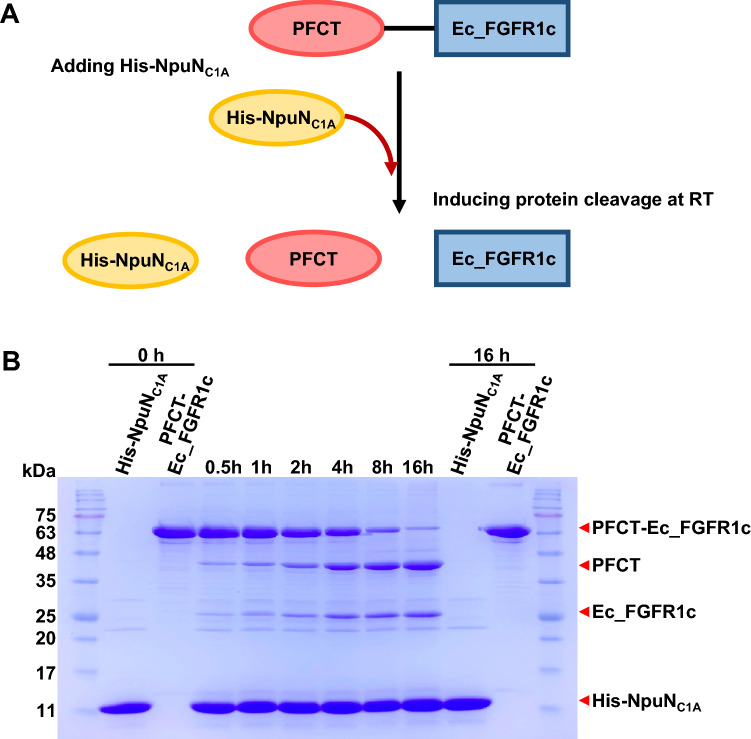
Schematic representation of intein-mediated cleavage reaction performed to separate fibroblast growth factor receptor (FGFR) from its fusion partner protease-free cleavable tag (PFCT) (**A**) and kinetic analyses of the cleavage reaction (**B**). After the purified PFCT-Ec_FGFR1c and His-NpuN^C1A^ proteins were mixed at a molar ratio of 1:5, the cleavage reaction was monitored in a time-dependent manner using SDS-PAGE. Contaminant proteins observed by SDS-PAGE in cleavage reaction were mainly originated from purified PFCT-Ec_FGFR1c. Red arrows indicate the whole and separated proteins. The added His-NpuN^C1A^ is also indicated by using an arrow.

The split intein-mediated protein trans-splicing occurs at a ratio of 1:1 between the N- and C-terminal fragments of the split intein^28^. To confirm the cleavage efficiency of PFCT, purified PFCT-Ec_FGFR1c and His-NpuN_C1A_ proteins were mixed at a ratio of 1:1 or 1:5 in PBS (pH 7.4) supplemented with 10 mM DTT at room temperature. In a previous report^28^, the trans-splicing efficiency under both molar ratios was time-dependent and reached a value of 85% after incubation for 16 h. However, our result showed that the cleavage reaction was independent of the molar ratio of the two proteins and their concentrations (Fig. 5 and S4). During repeated experiments, the cleavage efficiency of our system occasionally fluctuated in different batches of the purified proteins. These variations between experimental batches could be reduced to less than 5% by desalting the purified protein before the mixing for cleavage reactions. Considering that divalent cations such as Zn^2+^ could effectively inhibit intein-mediated protein splicing and cleavage^29,30^, divalent Ni^2+^ concurrently eluted from Histrap resins might hinder splicing reactions.

**Figure 6:**
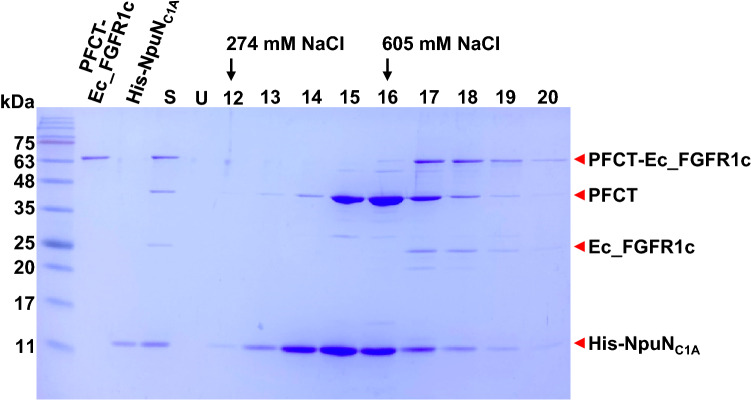
Elution profile analyses of the fibroblast growth factor receptor 1c (FGFR1c) isoform via affinity purification using heparin resin. As an intrinsic feature of binding affinity to heparin, the separated Ec_FGFR1c from PFCT-Ec_FGFR1c was eluted using approximately 605 mM NaCl. A similar elution profile was also shown by the PFCT-Ec_FGFR1c fusion protein that remained in the column. Red arrows indicate whole and separated proteins. His-NpuN^C1A^, which is the protein interacted with PFCT, was also co-eluted from the resin.

Although Ec_FGFR1c separated from the PFCT-Ec_FGFR1c fusion protein consisted of D2 and D3 domains without D1 and acid box, all conserved amino acid residues that interacted with heparin disaccharide in FGFR1c remained unchanged except for K160^21,31^. To verify the structural integrity of the separating Ec_FGFR1c, we further tried to purify this protein via affinity chromatography using a heparin column. After conduction of the intein-mediated cleavage reaction in 1.5 ml microtubes, the reaction solution was diluted with 50 mM sodium phosphate buffer (pH 6.5) and loaded into the heparin column. When eluted from the column with a linear gradient ranging from 0.1 to 1 M NaCl, His-NpuN_C1A_, PFCT, PFCT-Ec_FGFR1c and the separated Ec_FGFR1c were eluted at concentrations of approximately 274–605 mM NaCl (Fig. 6 and Fig. S5). According to the results obtained, two major unexpected proteins, His-NpuN_C1A_ and PFCT, could be separated from PFCT-Ec_FGFR1c and Ec_FGFR1c by washing steps with NaCl at appropriate concentrations (500–550 mM). This elution profile of Ec_FGFR1c was consistent with that of a previous report which stated that D1-D3 domains of FGFR1 were renatured using a dropwise or on-column refolding method and were then purified using heparin and anion exchange chromatography^12^. Although marginal fluctuations were observed during chromatography, approximately 40% or more Ec_FGFR1c was recovered, when compared with the used amount of PFCT-Ec_FGFR1c for intein-mediated cleavage. Although the cleavage reaction of Npu intein could aid the removal of a considerable proportion of PFCT from the PFCT-Ec_FGFR1c fusion protein under the specified conditions, the remaining PFCT-Ec_FGFR1c was concurrently eluted with the separated Ec_FGFR1c through heparin chromatography. These results implied that the Ec_FGFR1c protein in the separated and PFCT-fused state possessed a similar conformation and the same binding affinity to heparin. When needed, a different approach was applied to further separate the cleaved Ec_FGFR1c from uncleaved fusion protein PFCT-Ec_FGFR1c (Fig. S6).

FGFRs have been considered a promising druggable target in cancer or other diseases; however, recombinant FGFRs are produced mainly in eukaryotic systems with mammalian cells as hosts or via a post-refolding step using inclusion bodies. Using the recombinant *E. coli* system in the present study, we provided an alternative method for the soluble expression and separation of functional FGFRs by adopting a systematic approach using PFCT with dual functions (soluble expression and protease-free cleavage). When this tag was subjected to fusion, Ec_FGFR1-3, except for Ec_FGFR4, were mainly expressed as soluble proteins in *E. coli* and specifically separated via intein-mediated cleavage reaction of Npu intein. The affinity chromatography results using a heparin column and the consideration of the interaction of both proteins between MBP-Ec_FGFR1c and FGF1 strongly suggested that the separated Ec_FGFR1c from PFCT-fused proteins was functional. However, the structural integrity-based function of the resulting Ec_FGFR1c should be further clarified by conducting surface plasmon resonance interaction using its cognate ligand FGF1. Additionally, considering the structural similarity of other FGFRs to FGFR1c, PFCT is expected to be useful for the expression and separation of other FGFR proteins. Nevertheless, PFCT also presented with a few issues. For example, amino acid preference and additionally incorporated amino acids for the cleavage of the fused target protein^32–34^, especially around the fused position, could cause trouble. The loss of the target protein and thus its low recovery yield after intein-mediated cleavage should be further improved.

## Materials and methods

### Bacterial strain and plasmids

*E. coli* XL1-Blue (Δ(*mcrA*)*183* Δ(*mcrCB-hsdSMR-mrr*)*173 endA1 supE44 thi-1 recA1* gyrA96 relA1 lac *[F′* proAB lacI^q^*ZΔ*M15 *Tn*10 (*Tet*^*r*^)]) and BL21(DE3) (F^–^ *ompT* *gal* *dcm* *lon* *hsdS*_*B*_(*r*_*B*_^–^*m*_*B*_^–^) λ(DE3 [*lacI* *lacUV5*-*T7p07* *ind1* *sam7* *nin5*]) [*malB*^+^]_K-12_(λ^S^)) were used as the cloning and expression host, respectively. Recombinant vectors pET24a-hFGFR1c, pET24a-hFGFR2b, pET24a-hFGFR2c, pET24a-hFGFR3b, pET24a-hFGFR3c, pET24a-hFGFR4, and pET24a-hFGF1 were kindly gifted by Dr. Lee, Jung-Hyun (Korea Institute of Ocean Science & Technology, Korea) and used as templates for the amplification of *Ec_FGFR* genes by PCR. The DNA sequence of split intein *Npu*_PCC73102 was synthesized and provided by Bioneer Inc. (Daejon, Korea). pMAL-c2X (New England Biolabs, UK) and pQE30 (Qiagen, Germany) were used as plasmids for cloning PFCT, PFCT-Ec_FGFR fusion proteins, sole FGFRs, and NpuN_C1A_ gene, respectively.

### Cloning and construction of PFCT for FGFR production

DNA manipulations performed for the cloning and subsequent construction of PFCT were conducted based on the standard protocols generally used. Primer pairs for gene cloning and fusion protein construction are listed in Table 1. For the cloning of Ec_FGFR alone and MBP fused-Ec_FGFR proteins, the gene encoding Ec_FGFRs was amplified from pET24a-FGFRs by PCR using the corresponding primer pairs (Table 1) and high fidelity Phusion polymerase (New England Biolabs, USA). Thereafter, the PCR product was incorporating into the vectors pQE30 and pMAL-c2X subjected to digestion with *Bam*HI and *Hind*III, leading to the generation of pQE30-Ec_FGFR1c, pQE30-Ec_FGFR2b, pQE30-Ec_FGFR2c, pQE30-Ec_FGFR3b, pQE30-Ec_FGFR3c, pQE30-Ec_FGFR4, pMAL-Ec_FGFR1c, pMAL-Ec_FGFR2b, pMAL-Ec_FGFR2c, pMAL-Ec_FGFR3b, pMAL-Ec_FGFR3c, and pMAL-Ec_FGFR4 (Table S1). For the construction of PFCT, C- and N-terminal domains of Npu DnaE split intein were amplified from the synthesized *Npu*_PCC73102 with corresponding primer pairs in Table 1. The amplified C- and N-terminal domains were incorporated into the vector pMAL-c2X digested with *Sac*I and *Bam*HI and pQE30 digested with *Bam*HI and *Hind*III, leading to generation of pPFCT and pQE30-NpuN_C1A_, respectively. The N-terminal domain of Npu DnaE split intein was expressed as a recombinant protein with a 6× His tag to its N-terminus. To validate that the PFCT (Table S2) could be used for the production of functional FGFRs, Ec_FGFRs were cloned into the vector pPFCT by using the above-mentioned procedure, and pPFCT-Ec_FGFR fusion constructs were generated (Fig. 1).

**Table 1:** List of primers used in this study.

Primers	Sequence (5’ → 3’)	RE*
Ec_FGFR1c F	ATAGGATCCGAAAAGAAATTGCATGCAGTGCC	*Bam*HI
Ec_FGFR1c R	ATAAAGCTTTTACCTCTCTTCCAGGGCTTCCA	*Hind*III
Ec_FGFR2c/b F	ATAGGATCCGAAAAGCGGCTCCATGCTG	*Bam*HI
Ec_FGFR2b/c R	ATAAAGCTTTTACTCCTTTTCTCTTCCAGGCGC	*Hind*III
Ec_FGFR3b/c F	ATAGGATCCGACAAGAAGCTGCTGGCC	*Bam*HI
Ec_FGFR3b/c R	ATAAAGCTTTTACTCCACCAGCTCCTCCTC	*Hind*III
Ec_FGFR4 F	ATAGGATCCGAGAAGAAACTGCATGCAGTACC	*Bam*HI
Ec_FGFR4 R	ATAAAGCTTTTAGTCCTCCTCTGGCAGCAC	*Hind*III
QE_N-NpuC1A BamHI F	ATAGGATCCGCGACTAAAGCNCTGAGCTATG	*Bam*HI
QE_N-NpuC1A HindIII R	ATAAAGCTTTTAATTGGGCAGATTGTCAACGC	*Hind*III
PFCT_C-Npu SacI F	ATAGAGCTCGATCAAAATTGCGACCCGCAAG	*Sac*I
PFCT_C-Npu BamHI R	ATAGGATCCACTAGTCTTGTTAAAGCAGTTGCTTG	*Bam*HI

The target sequences incorporated in the primer for digestion with restriction enzymes have been underlined.

*Restriction enzyme used to digest the amplified DNA fragment by PCR with a set of specific primers.

### Analysis of expression patterns of Ec_FGFRs alone and MBP-fused Ec_FGFR proteins

To analyze the expression patterns of Ec_FGFRs alone and MBP-fused Ec_FGFR proteins, the recombinant *E. coli* XL1-Blue harboring pQE30-Ec_FGFRs or pMAL-Ec_FGFRs were streaked onto Luria Bertani (LB) agar plate supplemented with 100 µg/mL ampicillin and then grown at 37 °C. A single colony isolated from LB agar was seeded into 4 mL of LB broth containing 100 µg/mL ampicillin in a round-bottom tube (14 ml, Falcon) and incubated at 37 °C with constant shaking (220 rpm) conditions. After reaching an optical density (OD_600nm_) of 2.4–2.8, 1% (v/v) of the resulting seed culture was inoculated and cultured under the same conditions. After an optical density of approximately 0.5–0.6 at 600 nm (OD_600_) was achieved, 4 μL of 200 mM isopropyl β-D-1-thiogalactopyranoside (IPTG, final concentration of 0.2 mM) was added into the medium (4 ml culture volume of 14 ml round-bottom tube), and cells were further grown to induce protein expression for 3 h at 30 °C under the same conditions. The resulting culture was adjusted to an OD_600_ of approximately 2.0 and harvested by centrifugation at 16,100×g at 4 °C. Harvested cells were resuspended in 200 μL of phosphate buffered saline (PBS, pH 7.4) and then sonicated twice (2 s pulse on and 8 s pulse off) at 27% amplitude on ice (VCX 750, Sonic & Materials, USA). After performing lysis of the cells, the insoluble aggregates were removed by centrifugation at 16,100×*g* for 30 min at 4 °C. The total protein and soluble protein samples were obtained from cell lysates and supernatant after centrifugation, respectively. Aliquots of total and soluble proteins were mixed with a sample loading buffer (0.225 M Tris‐HCl pH 6.8, 50% glycerol, 5% SDS, 0.005 bromophenol blue, and 0.25 M DTT) at a 1:5 ratio, following which they were subjected to boiling for 15 min and resolution via SDS-PAGE using 12% gels. After electrophoresis, gels were stained with a Coomassie blue staining solution.

### Assessment of interaction test between MBP-fused Ec_FGFR1c and FGF1

To determine the interaction between MBP-Fused Ec_FGFRs and hFGF1, the recombinant *E. coli* XL1-Blue cells harboring pMAL-Ec_FGFR1c and BL21(DE3) harboring pET24a-FGF1 were streaked onto an LB agar plate supplemented with 100 µg/mL ampicillin and 50 µg/mL kanamycin, respectively, and were then subjected to growth at 37 °C. A single colony isolated from LB agar was seeded into 4 mL of LB broth containing antibiotics in a 14 ml round-bottom tube and incubated at 37 °C with constant shaking (220 rpm). After reaching an optical density (OD_600nm_) of 2.4–2.8, 1% (v/v) of the resulting seed culture was inoculated into 10 mL of LB broth supplemented with antibiotics and cultured under the same conditions. After an OD_600_ of approximately 0.5–0.6 was achieved, 10 μL of 200 mM IPTG at a final concentration of 0.2 mM was added into 10 ml of culture medium (50 ml Erlenmeyer flask), and the cells further cultured to induce protein expression for 3 h at 30 °C under the same conditions. Subsequently, the cultured cells were harvested. The resulting cultured *E. coli* XL1-Blue and BL21(DE3) cells harboring each construct were adjusted to an OD_600nm_ of 10 by resuspending in PBS (pH 7.4) and 50 mM sodium phosphate buffer (pH 6.5), respectively. After cells were disrupted by sonication under the same conditions described above, soluble lysates without insoluble aggregates were collected after centrifugation at 16,100×*g* for 20 min at 4 °C.

The fusion protein, MBP-Ec_FGFR1c, in the soluble lysates of *E. coli* XL1-Blue harboring pMAL-Ec_FGFR1c was collected using amylose resin (New England Biolabs, USA). 500 µL of the amylose resin was subjected to washing steps three times with 5 × the bed volume of PBS (pH 7.4) and then added into the soluble lysates. After mixing (rocking) for 60 min at 4 °C, the fusion proteins (MBP-Ec_FGFR1c) that bound to the amylose resin were collected by centrifugation at 1000×*g* for 1 min at 4 °C. Thereafter, the fusion proteins-bound amylose resins were washed twice with 5 × the bed resin volume of PBS and then with 5 × the bed resin volume of 50 mM sodium phosphate buffer (pH 6.5). Subsequently, the resulting MBP-Ec_FGFR1c-amylose resins were added into the soluble lysates (containing FGF1) of *E. coli* BL21(DE3) harboring pET24a-hFGF1, and they were then incubated for 60 min at 4 °C. Finally, FGF1 was eluted from MBP-Ec_FGFR1c-amylose resins by using a NaCl gradient with different concentrations (0.05, 0.2, 0.4, and 0.6 M) in 50 mM sodium phosphate buffer (pH 6.5). Eluted proteins in each fraction were analyzed by SDS-PAGE according to the general procedure.

### Analysis of expression patterns of PFCT-FGFR and His-NpuN_C1A_

Expression patterns of PFCT-Ec_FGFRs and His-NpuN_C1A_ were analyzed under the same conditions as described in a previous section ‘Analysis of expression patterns of Ec_FGFRs alone and MBP-fused Ec_FGFR proteins’**.**

### Purification of PFCT-Ec_FGFR fusion proteins and His-NpuN_C1A_

To prepare recombinant cells for protein purification, *E. coli* XL1-Blue was transformed with each construct (the PFCT-Ec_FGFR fusion protein and His-NpuN_C1A_) and plated on an LB agar plate containing 100 µg/mL ampicillin. After performing overnight culture, a single colony was selected and grown in 4 mL of LB broth containing the same antibiotics in a 14 ml round-bottom tube. After reaching an optical density (OD_600nm_) of 2.4–2.8, the resulting culture was further transferred to 300 mL of LB broth in a 1 L Erlenmeyer flask and was grown at 37 °C until an OD_600_ of 0.6 was achieved. Protein expression was induced at 30 °C for 3 h by the addition of IPTG at a final concentration of 0.2 mM. Subsequently, the induced cells were harvested by centrifugation at 6,000×*g* and 4 °C for 10 min.

To purify the recombinant proteins PFCT-Ec_FGFRs and His-NpuN_C1A_, harvested cells were rapidly frozen and slowly thawed twice before resuspension in 40 mL of PBS. After cell disruption via sonication (2 s pulse on 8 s pulse off for a total of 5 min at 40% amplitude on ice), insoluble aggregates were removed by performing centrifugation at 18,000×*g* and 4 °C for 30 min. Resulting supernatants containing the Ec_FGFR fusion protein and NpuN_C1A_ were loaded onto MBPtrap HP (1 mL, GE Healthcare Life Science, USA) and Histrap crude FF column (1 ml, GE Healthcare Life Science) pre-equilibrated with a PBS buffer (pH 7.4) at a flow rate of 1 ml/min at room temperature via fast performance liquid chromatography (GE Healthcare Life Science, AKTA Prime Plus FPLC system), respectively. The loaded MBPtrap HP and Histrap crude FF columns were washed extensively with PBS and PBS containing 35 mM imidazole until absorbance at 280 nm returned to baseline values, followed by elution with PBS containing 10 mM maltose and PBS containing 500 mM imidazole, respectively. The co-eluted contaminants including metal ions in the resulting solution of purified NpuN_C1A_ were removed by desalting using a Hitrap column (5 ml, GE Healthcare Life Science). The eluent was further subjected to intein-mediate cleavage reaction. The concentrations of purified proteins were determined by performing the Bradford assay using bovine serum albumin (BSA) as a standard.

### Soluble Ec_FGFR production via cleavage reaction of PFCT-Ec_FGFR using His-NpuN_C1A_

To remove RFCT from Ec_FGFR using His-NpuN_C1A_, purified PFCT-Ec_FGFR fusion proteins were mixed with His-NpuN_C1A_ without any pre-treatments under the specified conditions. Samples were collected at different time points after the initiation of the reaction, and were immediately subjected to boiling ed with 5× SDS sample buffer at 95 °C for 10 min; thereafter, they were analyzed using 15% SDS-PAGE gels. Band intensities corresponding to reactants and products were quantified using the ImageJ program (https://imagej.nih.gov/ij/download.html).

After the completion of cleavage reactions, Ec_FGFR1c was purified from the resulting solutions by considering the heparin-binding property of FGFR. The cleavage reaction solution was diluted five times with a dilution buffer (50 mM sodium phosphate buffer, pH 6.5), and was then loaded onto the HiTrap™ Heparin HP Column (Cytiva, USA) pre-equilibrated with dilution buffer. Thereafter, the columns were completely washed to remove undesired proteins and impurities with 50 mM sodium phosphate buffer (pH 6.5) containing 100 mM NaCl and then eluted using a linear gradient of 0.1–1 M NaCl. To separate uncleaved PFCT-Ec_FGFR1c, the resulting solution was further applied to MBPtrap column under general conditions described above. The purity of the eluted protein was also determined by SDS-PAGE.

## Supplementary Information

Supplementary Information.
