# Supplementary material for: A designed fusion tag for soluble expression and selective separation of extracellular domains of fibroblast growth factor receptors
Source: Sci Rep. 2021 Nov 2;11:21453. doi: 10.1038/s41598-021-01029-4 (PMC8563715; doi:10.1038/s41598-021-01029-4)
Supplement: Supplementary file 1 — Supplementary Information. [file 41598_2021_1029_MOESM1_ESM.docx]

**A designed fusion tag for soluble expression and selective separation of extracellular domains of fibroblast growth factor receptors**

Dea-Eun Cheong, Hye-Ji Choi, Su-Kyoung Yoo, Hun-Dong Lee and Geun-Joong Kim*

Department of Biological Sciences and Research Center of Ecomimetics, College of Natural Sciences, Chonnam National University, Yongbong-ro, Buk-gu, Gwangju, 61186, Korea.

Tel: +82-62-530-3403; Fax: +82-62-530-3409

*Correspondence should be addressed to:

Geun-Joong Kim

Department of Biological Sciences and Research Center of Ecomimetics, College of Natural Sciences, Chonnam National University, Yongbong-ro, Buk-gu, Gwangju, 61186, Korea

Tel: +82-62-530-3403; Fax: +82-62-530-3409; Email: gjkim@chonnam.ac.kr


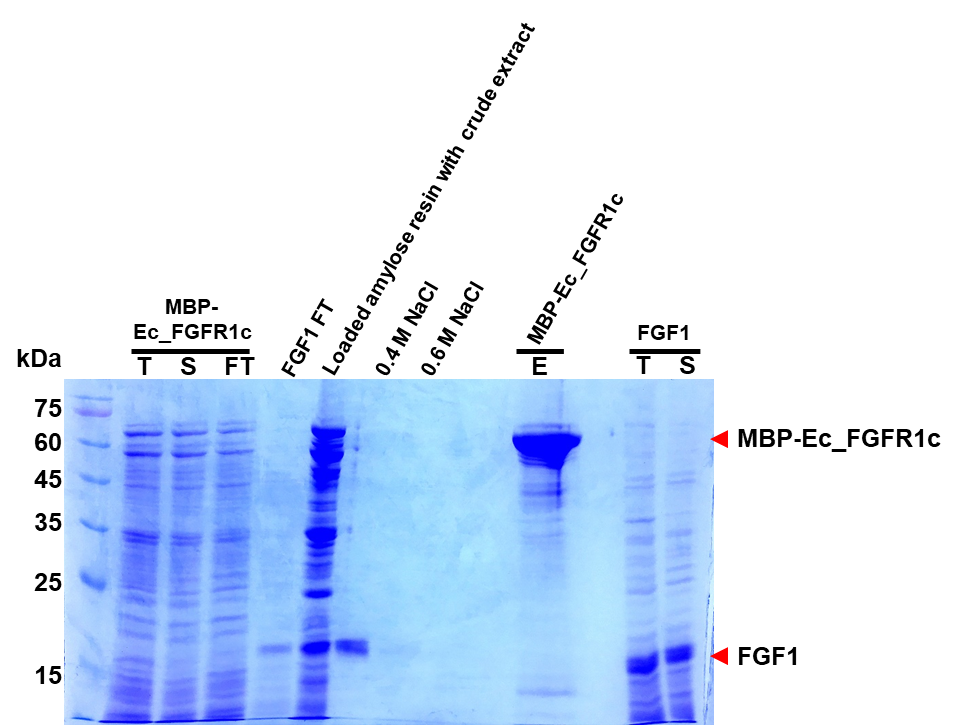


**Figure S1. Affinity purification of fibroblast growth factor 1 (FGF1) by functional receptor interaction using the amylose resin-bound MBP-Ec_FGFR1c**. The detailed purification procedure was described in the experimental section of the main text. An aliquot of the loaded amylose resin with the crude extract containing soluble FGF1 proteins was boiled and directly loaded into SDS-PAGE. The bound proteins were eluted with a buffer (50 mM sodium phosphate, pH 6.5) containing 0.4 and 0.6 M NaCl, respectively after washing with the same buffer (FGF1 FT) without NaCl. T, S, FT and E of MBP-Ec_FGFR1c denotes total, soluble, flow through and elution fraction, respectively. T and S of FGF1 denote total and soluble fraction, respectively.

**
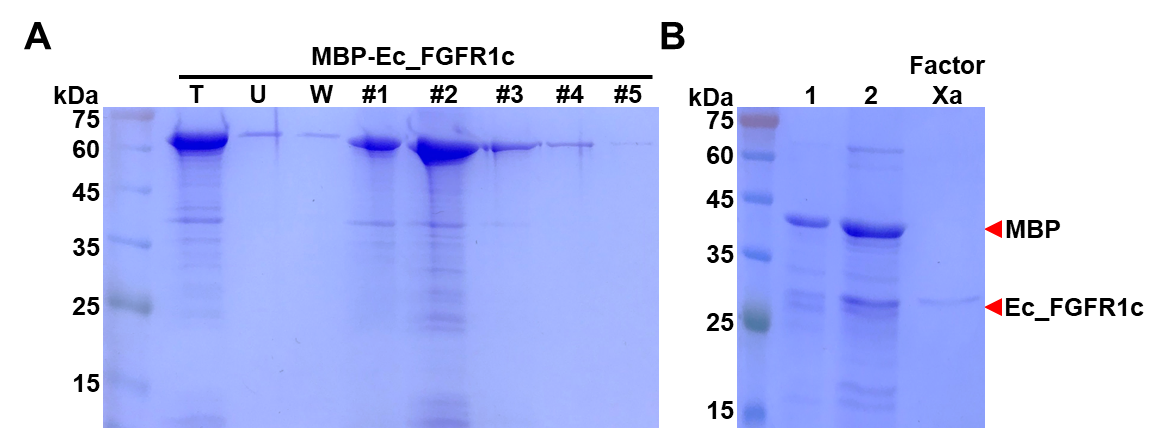
**

**Figure S2. Purification of maltose-binding protein (MBP)-fused fibroblast growth factor 1c (FGFR1c) fusion protein (A) and cleavage with Factor Xa (B)**. After the treatment of MBP-Ec_FGFR1c with Factor Xa, the corresponding bands to separated proteins, MBP and Ec_FGFR1c, were not clearly detected in SDS-PAGE due to non-specific degradation mediated by Factor Xa and/or contaminated proteases. T, U, W, and # denote total, unbound, washing and elution fraction, respectively.


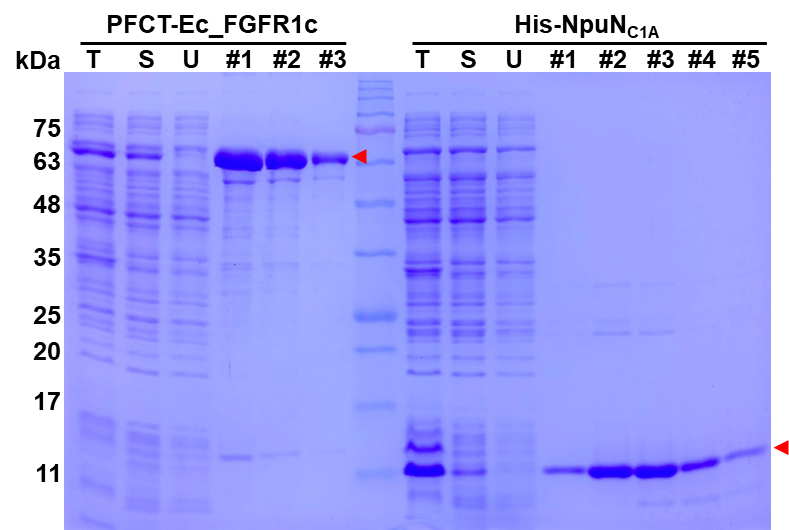


**Figure S3. SDS-PAGE analyses of purified PFCT-Ec_FGFR1c and His-NpuN_C1A_ proteins.** Both proteins were successfully purified via affinity purification using the amylose and Ni-NTA resins, respectively. T, U, and # denote total, unbound and elution fraction, respectively. Red arrows indicate the target proteins obtained.


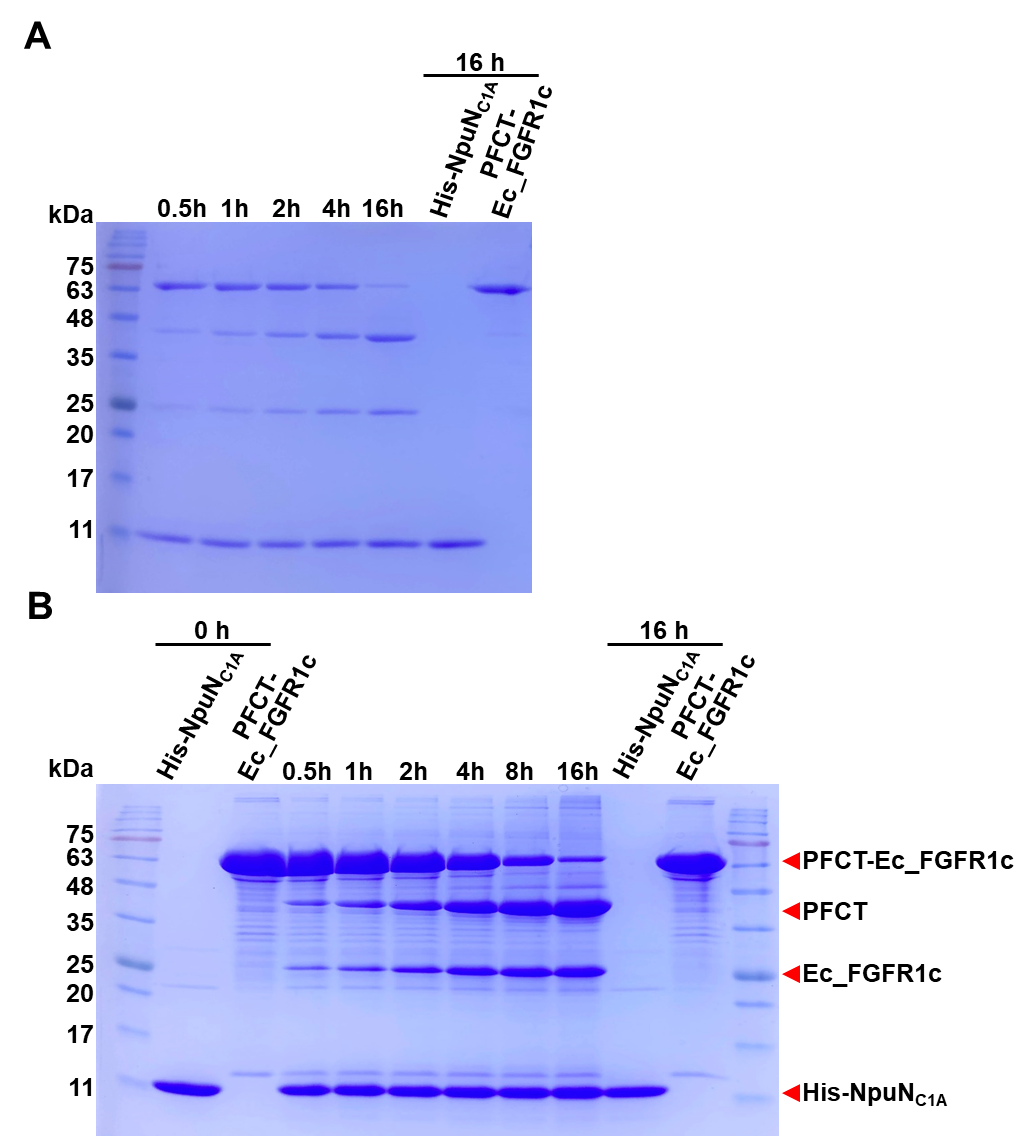


**Figure S4. Kinetic study of cleavage reaction with the different protein concentration (A) and ratio (B) under the same conditions of Fig. 5.** Kinetic analysis of panel A was performed under 1/10 reduced concentration of that used in Fig. 5. After the purified PFCT-Ec_FGFR1c and His-NpuNC1A proteins were mixed at a molar ratio of 1:1, the cleavage reaction was monitored in a time-dependent manner under the same condition of Fig. 5. Both profiles suggested that the cleavage reaction had no apparent dependency on protein concentration and molar ratio.

**
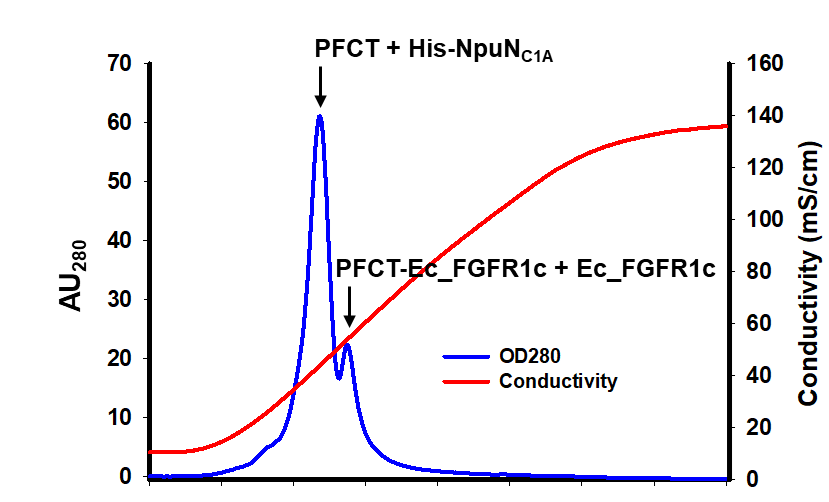
**

**Figure S5. Elution profiles of whole (PFCT-Ec_FGFR) and separated proteins via intein-mediated cleavage from the heparin resin**. The loading and then elution conditions were described in experimental section. As shown in this figure, whole and separated proteins were mainly co-eluted as mixtures in split peak due to protein-protein interaction.


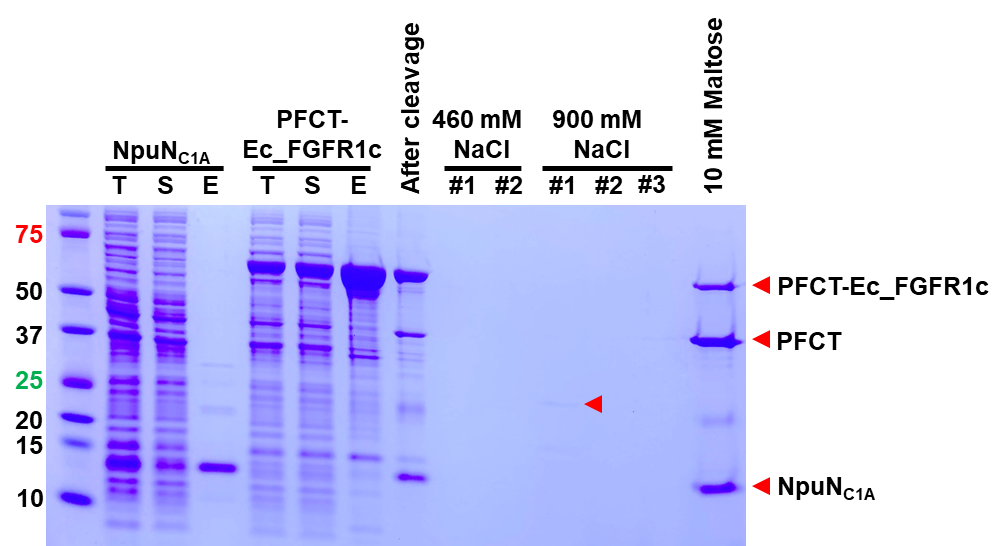


**Figure S6. SDS-PAGE analysis of the purified fraction containing Ec_FGFR1c**. The purification of His-NpuN_C1A_ and PFCT-Ec_FGFR1c, and intein-mediated cleavage reaction were conducted by procedures described in the experimental section of the main text. After the cleavage reaction of PFCT-Ec_FGFR1c by His-NpuN_C1A_ for 8 h, the resulting solution was diluted five times with a dilution buffer (50 mM sodium phosphate buffer, pH 6.5), followed by continuous loading onto consecutively connected two columns (1 ml HiTrap^TM^ Heparin HP and 1 ml MBPtrap column). The connected columns were pre-equilibrated with a dilution buffer at a flow rate of 1 ml/min at room temperature via fast performance liquid chromatography (GE Healthcare, AKTA Prime Plus). After completion of loading, connected columns were heavily washed with at least 200 ml of a dilution buffer to remove maltose from the binding pocket of MBP at a flow rate of 1 ml/min. Subsequently, nonspecifically bound proteins and other contaminants were washed with a dilution buffer containing 460 mM NaCl. Then, Ec_FGFR1c was eluted with a dilution buffer containing 900 mM NaCl. Finally, proteins bound to MPBtrap column were eluted with 10 mM maltose in a dilution buffer. The fraction eluted with 900 mM NaCl showed separated Ec_FGFR1c from uncleaved fusion protein (the corresponding band to Ec_FGFR1c was faint and also slightly retarded in diluted high salt conditions). An unknown protein was also observed in the eluted fraction containing Ec_FGFR1c. T, S, E and # denote total, unbound, washing and elution fraction, respectively.


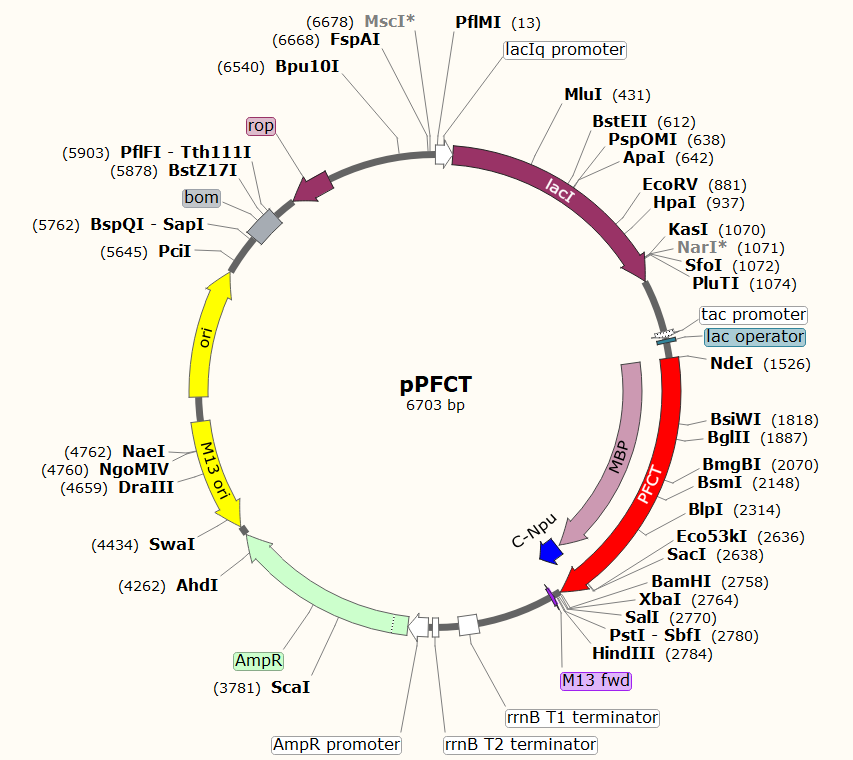


**Figure S7. Schematic representation of pPFCT vector map used in this work.**

Table S1. List of whole amino acid sequences of Ec_FGFRs used in this work for soluble expression

| Ec_FGFR | Amino acid sequence |
| --- | --- |
| FGFR1c | EKKLHAVPAAKTVKFKCPSSGTPNPTLRWLKNGKEFKPDHRIGGYKVRYATWSIIMDSVVPSDKGNYTCIVENEYGSINHTYQLDVVERSPHRPILQAGLPANKTVALGSNVEFMCKVYSDPQPHIQWLKHIEVNGSKIGPDNLPYVQILKTAGVNTTDKEMEVLHLRNVSFEDAGEYTCLAGNSIGLSHHSAWLTVLEALEER |
| FGFR2b | EKRLHAVPAANTVKFRCPAGGNPMPTMRWLKNGKEFKQEHRIGGYKVRNQHWSLIMESVVPSDKGNYTCVVENEYGSINHTYHLDVVERSPHRPILQAGLPANASTVVGGDVEFVCKVYSDAQPHIQWIKHVEKNGSKYGPDGLPYLKVLKHSGINSSNAEVLALFNVTEADAGEYICKVSNYIGQANQSAWLTVLPKQQAPGREKE |
| FGFR2c | EKRLHAVPAANTVKFRCPAGGNPMPTMRWLKNGKEFKQEHRIGGYKVRNQHWSLIMESVVPSDKGNYTCVVENEYGSINHTYHLDVVERSPHRPILQAGLPANASTVVGGDVEFVCKVYSDAQPHIQWIKHVEKNGSKYGPDGLPYLKVLKAAGVNTTDKEIEVLYIRNVTFEDAGEYTCLAGNSIGISFHSAWLTVLPAPGREKE |
| FGFR3b | DKKLLAVPAANTVRFRCPAAGNPTPSISWLKNGREFRGEHRIGGIKLRHQQWSLVMESVVPSDRGNYTCVVENKFGSIRQTYTLDVLERSPHRPILQAGLPANQTAVLGSDVEFHCKVYSDAQPHIQWLKHVEVNGSKVGPDGTPYVTVLKSWISESVEADVRLRLANVSERDGGEYLCRATNFIGVAEKAFWLSVHGPRAAEEELVE |
| FGFR3c | DKKLLAVPAANTVRFRCPAAGNPTPSISWLKNGREFRGEHRIGGIKLRHQQWSLVMESVVPSDRGNYTCVVENKFGSIRQTYTLDVLERSPHRPILQAGLPANQTAVLGSDVEFHCKVYSDAQPHIQWLKHVEVNGSKVGPDGTPYVTVLKTAGANTTDKELEVLSLHNVTFEDAGEYTCLAGNSIGFSHHSAWLVVLPAEEELVE |
| FGFR4 | EKKLHAVPAGNTVKFRCPAAGNPTPTIRWLKDGQAFHGENRIGGIRLRHQHWSLVMESVVPSDRGTYTCLVENAVGSIRYNYLLDVLERSPHRPILQAGLPANTTAVVGSDVELLCKVYSDAQPHIQWLKHIVINGSSFGADGFPYVQVLKTADINSSEVEVLYLRNVSAEDAGEYTCLAGNSIGLSYQSAWLTVLPEED |

Table S2. DNA and protein sequences of the designed construct PFCT for soluble cleavage reaction

|  | Sequence |  |
| --- | --- | --- |
| DNA | ATGAAAATCGAAGAAGGTAAACTGGTAATCTGGATTAACGGCGATAAAGGCTATAACGGTCTCGCTGAAGTCGGTAAGAAATTCGAGAAAGATACCGGAATTAAAGTCACCGTTGAGCATCCGGATAAACTGGAAGAGAAATTCCCACAGGTTGCGGCAACTGGCGATGGCCCTGACATTATCTTCTGGGCACACGACCGCTTTGGTGGCTACGCTCAATCTGGCCTGTTGGCTGAAATCACCCCGGACAAAGCGTTCCAGGACAAGCTGTATCCGTTTACCTGGGATGCCGTACGTTACAACGGCAAGCTGATTGCTTACCCGATCGCTGTTGAAGCGTTATCGCTGATTTATAACAAAGATCTGCTGCCGAACCCGCCAAAAACCTGGGAAGAGATCCCGGCGCTGGATAAAGAACTGAAAGCGAAAGGTAAGAGCGCGCTGATGTTCAACCTGCAAGAACCGTACTTCACCTGGCCGCTGATTGCTGCTGACGGGGGTTATGCGTTCAAGTATGAAAACGGCAAGTACGACATTAAAGACGTGGGCGTGGATAACGCTGGCGCGAAAGCGGGTCTGACCTTCCTGGTTGACCTGATTAAAAACAAACACATGAATGCAGACACCGATTACTCCATCGCAGAAGCTGCCTTTAATAAAGGCGAAACAGCGATGACCATCAACGGCCCGTGGGCATGGTCCAACATCGACACCAGCAAAGTGAATTATGGTGTAACGGTACTGCCGACCTTCAAGGGTCAACCATCCAAACCGTTCGTTGGCGTGCTGAGCGCAGGTATTAACGCCGCCAGTCCGAACAAAGAGCTGGCAAAAGAGTTCCTCGAAAACTATCTGCTGACTGATGAAGGTCTGGAAGCGGTTAATAAAGACAAACCGCTGGGTGCCGTAGCGCTGAAGTCTTACGAGGAAGAGTTGGCGAAAGATCCACGTATTGCCGCCACTATGGAAAACGCCCAGAAAGGTGAAATCATGCCGAACATCCCGCAGATGTCCGCTTTCTGGTATGCCGTGCGTACTGCGGTGATCAACGCCGCCAGCGGTCGTCAGACTGTCGATGAAGCCCTGAAAGACGCGCAGACTAATTCGAGCTCGATCAAAATTGCGACCCGCAAGTATCTGGGCAAGCAGAACGTGTACGATATTGGGGTGGAACGCGACCATAACTTTGCCCTGAAAAACGGTTTCATCGCAAGCAACTGCTTTAACAAGGGATCC | |
| Protein | MKIEEGKLVIWINGDKGYNGLAEVGKKFEKDTGIKVTVEHPDKLEEKFPQVAATGDGPDIIFWAHDRFGGYAQSGLLAEITPDKAFQDKLYPFTWDAVRYNGKLIAYPIAVEALSLIYNKDLLPNPPKTWEEIPALDKELKAKGKSALMFNLQEPYFTWPLIAADGGYAFKYENGKYDIKDVGVDNAGAKAGLTFLVDLIKNKHMNADTDYSIAEAAFNKGETAMTINGPWAWSNIDTSKVNYGVTVLPTFKGQPSKPFVGVLSAGINAASPNKELAKEFLENYLLTDEGLEAVNKDKPLGAVALKSYEEELAKDPRIAATMENAQKGEIMPNIPQMSAFWYAVRTAVINAASGRQTVDEALKDAQTN**SSS**IKIATRKYLGKQNVYDIGVERDHNFALKNGFIASNCF**NK** | |

Underlined letters indicate restriction enzyme (*Sac*I and *Bam*HI) recognition sites. Bold letters are encoded amino acids by nucleotide sequences that recognized by a restriction enzyme. Red letters indicate amino acid sequences of NpuC including three additional residues (+1 to +3) of natural extein.
